# Chronic Exertional Compartment Syndrome in the Forearm of a Collegiate Softball Pitcher

**DOI:** 10.1186/s40798-017-0080-5

**Published:** 2017-03-17

**Authors:** Austin Cole, John L. Hiatt, Christopher Arnold, Terry Sites, Ramon Ylanon

**Affiliations:** 1 University of Arkansas for Medical Sciences, 4301 W Markham St, Little Rock, AR 72205 USA; 2 University of Arkansas, Fayetteville, USA; 3 Advanced Orthopaedic Specialists, 3900 N. Parkview Dr, Fayetteville, AR 72703 USA

## Abstract

**Background:**

Chronic exertional compartment syndrome (CECS) is a recognized condition in the lower limb, with many reports in the literature. However, very few instances include CECS of the upper limb. This article presents the case of a collegiate softball pitcher presenting with CECS in her right forearm. To our knowledge, this is the first case report of a softball player with CECS, with only one similar incident in a major league baseball player.

**Purpose:**

The rarity of this condition normally places it low on the differential diagnosis. However, we hope that the presentation of this case and the review of the literature will aid in making swift and accurate diagnoses of CECS in future patients. We discuss the importance of three specific modalities in the diagnosis of this patient, what diagnostic criteria proved less conclusive, and the paradoxical course the syndrome presented with.

**Study Design:**

Case Review

**Results:**

Over a six-month period, the patient presented with peculiar presentations and exam results. A broad list of differential diagnoses had to be narrowed down through the presence or absence of relevant findings. These included cessation of exercise for 1 month, physical therapy, anti-inflammatory medicines, an electromyography/electromyogram (EMG), nerve conduction study (NCS), non-exercise magnetic resonance imaging (MRI) of the forearm, cervical MRI, and cervical computed tomography (CT) angiogram. After the above interventions were attempted and the relative findings of three important modalities were summarized, a fasciotomy and release of the dorsal, volar, and mobile wad compartments was performed. The patient’s symptoms were relieved, and she eventually returned to full play in softball at the university.

**Conclusions:**

The three diagnostic criteria we believed to be most helpful in this case, and for future cases of CECS in the forearm, include the clinical presentation, pre- and post-exercise MRI, and pre- and post-exercise compartment pressure measurements.

**Clinical Relevance:**

Chronic exertional compartment syndrome of the forearm is extremely rare, especially in the female athlete. This case report and review of the literature may be helpful to the clinician facing similar cases. It describes which clinical tests are most helpful for diagnosis and which findings may be distracting.

## Key Points

-An unusual case of chronic exertional compartment syndrome in the forearm of a female collegiate softball pitcher
-Recommendations developed for the work-up of chronic exertional compartment syndrome in the upper extremity
-A literature review of intracompartmental pressure measurement recommendations for diagnosing chronic exertional compartment syndrome in the forearm

## Introduction

Chronic exertional compartment syndrome (CECS) is classically defined as a condition presenting with recurrent, ephemeral increases in pressures of confined muscle compartments during exercise. Acute compartment syndrome of an extremity may develop from either traumatic intracompartmental swelling or external compression. However, CECS usually only presents with exercise of the affected compartment, and typically resolves with rest. Increased pressure within compartments leads to transient pain, paresthesia, numbness, and hindrance of muscle activity [1]. CECS is often overlooked as the cause of muscle pain and paresthesia in the extremities, due to its rarity and diverse manifestation. CECS may present with many different symptoms, appear identical to other etiologies, lack physical exam findings, and appear intermittently as well as transiently [2, 3]. Diagnosis can be delayed as long as 22 months in some instances [2].

The pathophysiology of CECS is not completely understood, but it is certainly different from the more familiar acute compartment syndrome. Rather than through abrupt injury, the pressure within the compartment involved rises upon exercise of the extremity and related muscles. The pathophysiology could possibly be a result of a combination between increased muscle size from higher blood flow, increased tension of the surrounding fascia, higher production of metabolic products, and an increase in extracellular water content [4, 5]. It is thought that expansion of the exercised muscle compartment can be up to 20% in volume [6].

Typically, CECS is seen in the young adult athlete who maintains a strict schedule of intense exercise. Most cases of CECS in literature are of the lower extremities, with only a few involving the forearm. Furthermore, almost the entirety of the upper extremity presentations is a result of either manual labor, rowing, motocross, weight lifting, or kayaking [5–11]. This article presents the case of a collegiate softball pitcher presenting with CECS in her right forearm. To our knowledge, this is the first case of a softball player with CECS in literature, with only one comparable case in a major league baseball player [6].

## Case Review

A 21-year-old female softball pitcher and outfielder presented with a chief complaint of right forearm pain and paresthesia. The patient described a burning, tingling sensation over the lateral portion of right forearm for the previous 4 weeks. Initially, symptoms presented only with extensive throwing and upper extremity exercise. However, by this visit it had progressed to presenting in the weight room and even occasionally at rest. She had tried activity modification through decreasing both duration and intensity workouts, along with over the counter anti-inflammatories. However, these did not relieve her discomfort. Physical exam showed full range of motion at the elbow and wrist with flexion, extension, pronation, supination, and radial and ulnar deviation. There was no sign of pigment changes, warmth, or erythema. Palpation revealed hypersensitivity over the lateral right forearm, tenderness over lateral epicondyle, and slight tenderness over medial epicondyle. Tinel’s sign was negative over the cubital tunnel, Adson’s sign was negative, and there was no epitrochanteric lymphadenopathy. A broad differential diagnoses at this point included peripheral nerve entrapment, peripheral neuropathy, motor neuron pathologies, and muscular disorders. Subsequently, the plan was to begin non-steroidal anti-inflammatory drugs (NSAIDs) and to order a nerve conduction study (NCS) and electromyography (EMG).

The NCS and EMG tests were normal, and the NSAIDs were not effective over a period of 4 weeks. Due to consistent and reproducible symptoms with lack of resolve, further work-up was pursued. With a speculation of previous injury, an anatomic abnormality, or compartment syndrome, an MRI of the right forearm was ordered. The result was also normal. With no conclusive evidence of compartment syndrome or soft tissue-related etiology of the forearm, the differential diagnosis shifted toward vascular and neurological etiologies in the cervical region. Thus, a cervical MRI was ordered. With the exception of a slightly increased T1 image finding in the right vertebral artery, there were no significant findings leading to a presumptive diagnosis of an underlying injury in the cervical region. To follow up on the right vertebral artery signal, a CTA of the neck was ordered, which was also found normal.

After weeks of studies, the patient still maintained symptoms. At a second clinical evaluation, the patient endorsed some tenderness at rest. The patient was instructed to exercise, during which she performed burpees, push-ups, and medicine ball tosses until pain and tightness in the forearm were felt (at 10 min). She then continued 5 min longer, totaling 15 min of exercise. Upon evaluation, the surgeon found discoloration of the right forearm, tenderness to touch over both epicondyles, and pain in the flexor and extensor compartments. The patient also presented with stiffness and a feeling of heavy pressure within the right forearm. Consequently, a bilateral pre-exercise and post-exercise MRI was ordered to work up possible exercise-induced compartment syndrome.

**Fig. 1:**
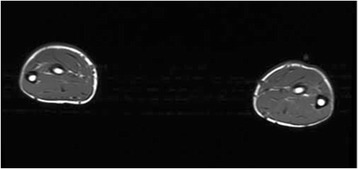
Bilateral forearm MRI pre-exercise

**Fig. 2:**
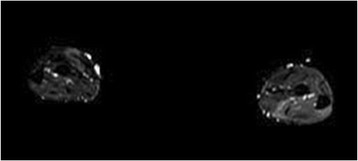
Bilateral forearm MRI post-exercise (symptomatic right arm, asymptomatic left arm)

The post-exercise MRI found abnormal muscle edema within the flexor and extensor compartments of the right forearm (Figs. 1 and 2). In the flexor compartment, edema presented within the flexor carpi radialis, flexor digitorum superficialis, and flexor carpi ulnaris muscles. Extensor edema was subtler, extending within the muscle belly of the extensor carpi radialis brevis and longus. The left forearm served as a control, with both pre- and post-exercise values found normal. In conclusion, radiographic findings were consistent with CECS of the right forearm, with greater involvement of the volar compartment than dorsal compartment.

The differential diagnosis at this point included CECS or an unusual presentation of nerve entrapment. The patient was informed and was selected to proceed with intramuscular pressure measurement over a further work-up of nerve entrapment. For definitive diagnosis of CECS, compartment pressure increase upon exercise was needed.

Pressure was checked three times: pre-exercise, 3 min post-exercise, and 5 min post-exercise. An injection of 1% lidocaine was used to achieve local pain reduction. A catheter was then introduced into the dorsal and volar mid-forearm areas of both the right and the left upper extremities. The transducer was attached. Pre-exercise pressure values were then obtained bilaterally.

**Fig. 3:**
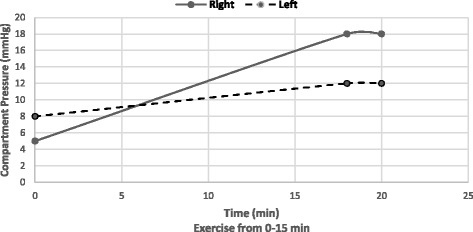
Bilateral dorsal compartment pressures

**Fig. 4:**
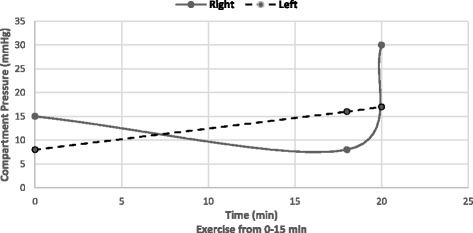
Bilateral volar compartment pressures

The patient then proceeded with exercise, with the identical plan as the exercise MRI work-up. She continued until symptoms arose (once again at 10 min), and then continued 5min longer for a total time of 15 min. Clinical examination revealed an exceptionally swollen and tight right forearm. There was no radiation to the hand or fingers. The symptomatic right forearm dorsal compartment measured 5 mmHg pre-exercise, and 18 mmHg at 3 and 5 min post-exercise. The symptomatic right forearm volar compartment measured 15 mmHg pre-exercise, 10 mmHg 3 min post-exercise, and 30 mmHg 5 min post-exercise. See Figs. 3 and 4 for comparison of pressures.

Following a full patient discussion and consultation, with the understanding that CECS of the forearm is an unusual presentation and difficult diagnosis, it was felt that these findings (along with the presentation and extensive work-up throughout the previous 6 months) were significant enough to proceed with right forearm fascial releases. An initial curvilinear incision was made across the antecubital fossa and extended downward on the right volar forearm. A fasciotomy was then completed, including release of the bicipital aponeurosis. Finally, careful palpation for any other tight bands was performed. Attention was then turned to the dorsal forearm. A longitudinal incision was made over the mid-dorsal forearm, and a fascial release was performed proximally and distally. The mobile wad was released as well. Copious irrigation was then performed. Tourniquet time totaled 17 min. Simple nylon closure of the incisions was performed, followed by a sterile compressive dressing. There were no noted complications before, during, or after surgery. The right forearm was then placed in a posterior splint until the dorsal wound healed.

The patient was seen in clinic 7 days post-compartment release of the forearm. The right hand was found swollen, but soft. The patient had a strong radial pulse and good wrist flexion and extension. Flexion measured 100°, and extension 60°. A postoperative hematoma had established under the dorsal wound, and the posterior splint was discontinued and was replaced with a sling. A nine-day assessment was made to evaluate the inflammation, at which the presentation and pain were much improved. At 13 days, the patient could make a full fist, pronation measured 90°, supination 90°, extension 80°, and flexion 150°. 15 ccs of blood was aspirated from the previously mentioned subcutaneous hematoma. At 6 weeks post-release, all compartments were soft, non-tender, and the wounds benign. The patient was cleared to begin rehabilitation. Specific exercises for the subsequent 2 months and an interval-throwing program were pursued. Slow progression into light weight, high repetition wrist and arm exercises was the first step. The athlete then began light overhand throwing of the softball. The patient progressed slowly to underhand throwing over time with no pain. The anti-inflammatories Relafin and Voltaren Gel were prescribed as she started to increase activity. At 4 months post-compartment release, she was cleared for full play without the use of any anti-inflammatories. See the timeline of events in Table 1.

**Table 1:** Case timeline

March 30	First presentation to a sports medicine physician.
April 2	EMG and NCS. Results normal.
May 26	Second presentation to orthopaedic surgeon with the same symptoms. Plan included 1 month of rest and a MRI.
May 28	MRI of right forearm and elbow. Results normal.
May 29	Second opinion from physician who agreed MRI was normal, and the presentation was not developed enough to indicate CECS.
June 3	MRI of cervical region to rule out neurological etiology.
June 4	Angiogram of cervical region, specifically right vertebral artery, to rule out vascular etiology.
June 4–September 1	Patient was home for the summer. Played no summer ball and rested the forearm.
September 1	Third presentation. The patient manifested pain upon exercise in clinic.
September 11	MRI of bilateral forearms pre- and post-exercise. Results found to be diagnostic of CECS.
September 14	Fourth presentation. Some findings unexpected.
September 16	Compartment pressure measurements pre- and post-exercise. Fasciotomy and release of right forearm dorsal, volar, and mobile wad compartments.
September 22	7 day follow-up. Symptoms greatly relieved, yet postoperatively swelling.
September 24	9 day follow-up. Swelling found subsiding.
September 29	13 day follow-up. Stitches removed and subcutaneous hematoma aspirated.
October 27	6 week follow-up. Forearm movement back to normal and wounds benign.
January 19	4 month follow-up.

## Discussion

This presentation of CECS in the forearm of a female collegiate softball pitcher is exceptionally unique. CECS has appeared in the upper extremity in perhaps only a few dozen instances in literature, usually in certain populations (soldiers, motocross, kayaking) [1, 4–8, 10–12]. In addition to the rarity, this case illustrates the difficulty of diagnosing CECS of the forearm. From the initial presentation and plan to acquire EMGs and a NCS to the normal neurological, vascular, and muscular work-ups, the diagnosis of CECS can be recognized as very challenging. In this section, we discuss three modalities we believe aid most in diagnosing CECS in the forearm, methods that have proven less effective, and the paradoxical course this particular case took.

The first of the three useful modalities is simply clinical presentation. Although this is not a true *modality* by definition, patient presentation through signs, symptoms, and physical examination proved just as vital to the diagnosis as any true modality would. Specifically, one should look for forearm pain and paresthesia over either the dorsal or the volar aspect of the forearm during exercise. Our patient presented with history of pain upon exercise in both forearm regions, described it as a “burning, tingling sensation”. Erythema, numbness, and hindrance of motion due to discomfort also accompanied the exertional pain. All these are characteristic of other cases of CECS [1–4, 6–10, 13]. Notably, the symptoms never radiated to the hand over the six-month course. Hypersensitivity to palpation over the lateral forearm, tenderness to palpation over lateral epicondyle, and slight tenderness over the medial epicondyle can be further indications of CECS. Another element to consider in future diagnoses is a possible vague, seemingly benign physical exam. The patient maintained full range of motion at the elbow and wrist with flexion, extension, pronation, supination, and radial and ulnar deviation when at rest. Furthermore, pigment changes, warmth, and erythema was only seen upon exercise. It is therefore very important to reproduce symptoms by specific exercise. However, unusual for CECS, our patient did have some history and one clinical exam where tenderness presented at rest. A lack of Tinel’s sign and Adson’s sign, as in this instance, might also aid in diagnosing CECS. Finally, the lack of anti-inflammatory relief could suggest a possible CECS case.

A second modality that proved to be extremely helpful in diagnosing CECS was the pre- and post-exercise MRIs. This style of imaging allows assessment of signal intensity changes over time to quantify fluid accumulation within soft tissues. Furthermore, MRIs are safe and prevent exposure to radiation. However, we believe it is important to ensure the history and clinical evaluations steer toward CECS (specifically reproducing symptoms with exercise) before ordering such costly studies. In addition to this case, pre- and post-exercise MRIs have been proven to aid in the CECS work-up elsewhere in literature [13–18].

The third and final modality we recommend is pre- and post-exercise intracompartmental pressure measurements. Because MRIs and clinical presentation are non-invasive techniques, we believe that the addition of compartment pressure evaluation is obligatory before a definitive diagnosis is made and a fasciotomy pursued. Although there has been evidence of variance in normal compartment values, any significant rise in pressure post-exercise is highly indicative of CECS [2, 3, 6, 12, 15, 17, 19]. In fact, many other studies have concluded that the best method for diagnosing CECS is evaluation of intracompartmental pressures (ICPs) before and after exercise [2, 5, 12, 15, 17, 20]. However, in agreement with Rorabeck et al., we believe ICPs are only a supplemental component to the history and physical examination. In conclusion, we recommend a work-up of forearm CECS using all three modalities.

First and foremost, it is important to understand what the literature proposes as *normal* for ICP values. The standard resting pressure in normal compartments is between 0–15 mmHg in the lower leg. Borderline pressures are from 16–24 mmHg, while values above 25 mmHg are consistent with the diagnosis of CECS. Even though these values are based on the much more common presentation of CECS in the lower limb, they are used in most studies of CECS in the upper extremity [2, 3, 15–17, 19]. Due to the rarity of upper extremity cases, Ardolino et al. attempted to experimentally determine normal, asymptomatic, forearm pressures before and after exercise [12]. With a 95% CI, normal, asymptomatic forearm extensor pressures in 41 volunteers were found to be between 0–25.3 mmHg, and normal forearm flexor pressures between 0–21.4 mmHg [12]. Interestingly, there was no significant difference between pre- and post-exercise values. Ardolino et al. also found resting and exercise numerical values significantly higher than what most forearm studies (using ICP values of the leg) had considered normal. [15–17, 19]. In fact, the 95% CI, 0–25.3 mmHg dorsal ICP resting range surprisingly includes the theoretical diagnostic value of CECS (20 mmHg) used in so many other studies. In conclusion, we believe the accuracy of normal and resting ICPs that many studies provide for the forearm is questionable, with a wide variance [12, 17, 19, 20].

With normal values proving inconsistent in literature, we now look at what studies recommend for diagnosing CECS with ICP values. A hypothetical diagnostic criterion for CECS in the forearm has been proposed by Pedowitz et al.; a resting ICP of 15 mmHg or greater, a 1–3-min post-exercise ICP of 30 mmHg or greater, and a 5-min post-exercise ICP of 20 mmHg or greater. Of note, this has only been tested with compartments of the leg [1, 2, 12, 17].

In comparison to the *diagnostic criterion*, our patient’s resting symptomatic arm measured 5 mmHg in the dorsal compartment and 15 mmHg in the volar [17]. Just the dorsal compartment ICP barely met the *diagnostic* value of 15 mmHg that Pedowitz et al. recommends, and neither ICP was above the upper end of *normal* (25.3 mmHg) proposed by Ardolino et al. in the 2010 forearm pressure study [12, 17]. In agreement with Roscoe et al, we decided to extend exercise 5 min from the onset of pain in order to build pressure and edema enough to allow a cushion of time for inserting the catheters. At 3 min post-exercise, the dorsal ICP rose 13 mmHg to a value of 18 mmHg, while the volar ICP actually dropped 5 mmHg to a value of 10 mmHg. Once again, this is contrary to what literature recommends: 30 mmHg at 3 min post-exercise for a CECS diagnosis [12, 17, 20]. However, at 5 min post-exercise, the dorsal compartment maintained its 18 mmHg value, while the volar compartment rose to 30 mmHg. As a very important conclusion, the dorsal resting ICP and the volar 5-min post-exercise ICP were the only values of the six taken that fit in the *diagnostic criterion* for CECS proposed by Pedowitz et al. See Table 2 for ICP value comparison.

**Table 2:** ICP value comparison

Measurement	Ardolino et al. proposed abnormal value	Pedowitz et al. proposed CECS diagnostic value	Current case actual value
Pre-exercise volar	>21.4 mmHg	>15 mmHg	15 mmHg
Pre-exercise dorsal	>25.3 mmHg	>15 mmHg	5 mmHg
3 min. post-exercise volar	>21.4 mmHg	>30 mmHg	10 mmHg
3 min. post-exercise dorsal	>25.3 mmHg	>30 mmHg	18 mmHg
5 min. post-exercise volar	>21.4 mmHg	>20 mmHg	30 mmHg
5 min. post-exercise dorsal	>25.3 mmHg	>20 mmHg	18 mmHg

Note: Ardolino et al. concluded that there was no significant difference in pre- and post-exercise values [12]

Because of conflicting studies, variance in results, and contrasting data with this patient, we believe that utilizing a particular numerical value for ICP is not helpful. Instead, the important factor for CECS seems to be the rise in pressure from resting to 5 min after exercise, perhaps as a percent, and delayed normalization. All reports in literature support that as a consistent phenomenon [1, 2, 12, 17, 20]. Perhaps these two elements should be investigated more thoroughly in future studies.

Unlike the three above modalities we believe are useful in diagnosing CECS of the forearm, other choices have often proven inadequate. As in this case, observation with cessation of exercise for 1 month, a physical therapy plan, anti-inflammatory medicines, an EMS, a NCS, a non-exercise MRI of the forearm, a cervical MRI, and a cervical CT angiogram have all demonstrated to only rule out a few differential diagnoses or have proven inconclusive. Even though the first step taken by the initial physician in March was to rule out any concomitant nerve compression through EMG/NCS studies, these exams may prove misleading or inaccurate at times [15, 21–24]. Other investigations not done in this case have proven futile as well; including X-rays, blood tests, and Doppler ultrasound scans [2, 13].

A final interesting component of this case is the paradoxical presentations throughout the 6 months. The September 14 visit, after the exercise MRI, delivered unexpected findings. First, unlike the earlier examinations, there was no tenderness to palpation or pain with rest. The patient stated that now only with exercise she experienced discomfort, swelling, erythema, and numbness. However, this constituted only minimal exercise; manifesting after just 30 s, and even through handwriting. Second, during this visit, the pain was specifically over the dorsal compartment, with less in the volar region. This seems contrary to the MRI findings just 3 days prior, where abnormal edema was primarily found within the flexor carpi radialis, flexor digitorum superficialis, and flexor carpi ulnaris muscles. It is possible that the cessation of softball and strenuous activity over the two-month summer period in July and August alleviated some of the symptoms. However, it still is important to understand that CECS may present differently at subsequent visits.

## Conclusions

Due to the extensive clinical examinations and diagnostic work-ups, the successful diagnosis of CECS was finally made in this case. If the three modalities of clinical presentation, exercise MRIs, and ICP measurements had already been established as a step by step protocol, the diagnosis of CECS would have been accomplished much sooner. In review of this case, we believe these three modalities are most important and should be considered in future cases of CECS work-up. Furthermore, we recommend a case-control study examining percent rise from pre- to post-exercise compartment pressures and the time delay in recovery. Finally, the surgical intervention of CECS in the forearm seems to be necessary, since therapy, one-month cessation of exercise, and anti-inflammatories did not prove curative. This case presented over a six-month period without any resolution using these techniques (Table 1). Surgical fasciotomy is encouraged highly in literature for CECS and is proved to be the definitive solution once CECS was diagnosed [2, 7, 10, 11, 14, 19, 20, 25–29]. To our knowledge, this is the first report of a case of chronic exertional compartment syndrome in the forearm of a collegiate softball pitcher. The rarity of this condition normally places it low on the differential diagnosis, however, we hope that the conclusions of this case will aid in making swift and accurate diagnoses of upper extremity CECS in future patients.
